# The Pathogenesis of Human Cervical Epithelium Cells Induced by Interacting with *Trichomonas vaginalis*

**DOI:** 10.1371/journal.pone.0124087

**Published:** 2015-04-22

**Authors:** Wei-Chen Lin, Wei-Ting Chang, Tsuey-Yu Chang, Jyh-Wei Shin

**Affiliations:** Department of Parasitology, National Cheng Kung University, Tainan, Taiwan, Republic of China; Georgetown University, UNITED STATES

## Abstract

**Background:**

*Trichomonas vaginalis* is a protozoan parasite that occurs in the urogenital-vaginal tract and is the primary causative agent of trichomoniasis, a common sexually transmitted disease in humans. The aggregation of this protozoan tends to destroy epithelial cells and induce pathogenesis.

**Principal Findings:**

This study cultured *T*. *vaginalis* and human cervical epithelial cells (Z172) under the same conditions in the experiments. Following co-culturing for ten hours, the protozoans became attached to Z172, such that the cells presented a round shape and underwent shrinkage. Time-lapse recording and flow cytometry on interacted Z172 revealed that 70% had been disrupted, 18% presented a necrosis-like morphology and 8% showed signs of apoptosis. Gene expression profiling revealed in the seven inflammatory Z172 genes as well as in *T*. *vaginalis* genes that code for adhesion proteins 65 and 65-1.

**Significance:**

These results suggest that cytopathogenic effects progress while Z172 is in contact with *T*. *vaginalis*, and the resulting morphological changes can be categorized as disruption.

## Introduction

*Trichomonas vaginalis*, an anaerobic and flagellated parasitic protozoan, is a causative agent of trichomoniasis, one of the most common sexually transmitted disease (STD) [1]. In men, this infection is usually asymptomatic; however irritating urethritis or prostatitis occurs in a few cases. In women, the disease is associated with a wide spectrum of symptoms, ranging from a relatively asymptomatic state to severe inflammation [2]. Ten percent of infections present with a "strawberry cervix" or vagina on examination. Previous studies have reported that the presentation of *T*. *vaginalis* increases the risk of acquiring HIV [3–6]. A great deal is known about trichomoniasis; however, the pathogenic mechanisms and influences of host-parasite interactions remain largely undefined. Adhesion is believed to play an important role in the onset of infection and the *T*. *vaginalis* cytolysis of human epithelial cells is contact dependent [7–11]. Adhesion of *T*. *vaginalis* to red blood cells [10], or those of the prostate or ectocervical epithelium [11] triggers the degradation of the host cell membrane skeleton, which can lead to cytolysis.

Previous research into the molecular aspects of *T*. *vaginalis* adhesion to human cells has identified a number of adhesion molecules on the surface of the parasite, the proteins of which include AP65, AP51, AP33, and AP23 [12, 13]. Among these, AP65 is the prominent protein mediating the binding of parasites to host epithelial cells [14–17]. Little is known about the molecules binding the parasites to the host cell receptors; however, a growing body of evidence suggests that Laminin may be a target in trichomonad adhesion [18–20]. The only host cell receptor of *T*. *vaginalis*, galectin-1, has been identified on cervical epithelial cells and has been shown to bind to *T*. *vaginalis* lipoglycan (TVLG) [21].

The *T*. *vaginalis* trophozoites colonize and parasitize the vagina or prostate of infected hosts. The trophozoite divides by binary fission and, in natural infections, gives rise to a population in the lumen and on the mucosal surfaces of the urogenital tracts of humans. The trophozoite is oval and flagellated; however, the parasite develops an amoeboid morphology when adhering to epithelial cells [22]. As the parasite multiplies in it’s amoeboid stage, it attaches to the squamous epithelium in the genital tract, causing inflammation of the vagina. During menstruation, the environment of the vagina changes, resulting in an increase in the severity of clinical symptoms and the rapid growth of protozoa. The overall immune response during trichomoniasis is largely unknown and high levels of interleukin-8 (IL-8) [23] and leukotreine B4 (LTB4) have been identified in the vaginal secretions of patients symptomatic with trichomoniasis [24–27].

This study established a co-culture system to investigate the interaction of *T*. *vaginalis* with the human cervical epithelial cells, Z172, in order to gain a deeper understanding of the mechanism underlying the pathogenesis of *T*. *vaginalis*. Our aim was to reveal the alterations that occur in morphology and gene expression due to the interactions between *T*. *vaginalis* and epithelial cells.

## Methods

### Cell culture

Trichomonas vaginalis strain ATCC 30236 (JH31A, USA), were cultured axenically at 37°C in iron free Yeast extract, Iron-Serum (YI-S) medium [28] supplied with 2% haemoglobin and medium supplemented with 10% Nu-serum. Organisms grew to mid-log phase were used in the subsequent experiments. Human cervical epithelial cells (Z172) was culture in pH 7.2 low glucose Dulbecco's modified Eagle's medium (DMEM), which supplemented with 10% Nu-serum and 2% hemoglobin bovine at 37°C incubator in the presence of 5% CO_2_. After adaptation of the *T*. *vaginalis* and Z172 cells were cultured in the same culture condition (DMEM-YI-S medium (2:1, vol/vol)).

### Adaptation of *T*. *vaginalis* and Z172

#### Trichomonas vaginalis

Addition DMEM medium in YI-S medium have been in continuous culture for 20 serial passages arrives at DMEM-YI-S medium. Medium joins the half new DMEM: YI-S-2:1 medium. Finally, *T*. *vaginalis* were cultured in the DMEM-YI-S medium (2:1, vol/vol) and supplied with 2% haemoglobin.

#### Z172 cell

Addition YI-S medium in DMEM medium have been in continuous culture for serial passages in the same culture condition DMEM-YI-S medium. Medium joins the half new DMEM-YI-S medium (2:1, vol/vol) and supplied with 2% haemoglobin.

### Growth curves

*T*. *vaginalis* were cultured in the YI-S and DMEM-YI-S medium (2:1, vol/vol) supplied with 2% haemoglobin. Detached cells were centrifuged and the pellet was resuspended in a 0.5% trypan blue solution in sterile PBS for one min. Cells were then counted in a neubauer hemocytometer chamber, and the stained cells were considered dead. Cell counting was performed under light microscope every six hours in the duration of forty-two hours to get the numbers of alive and dead cell, which were differentiated by the trypan blue dye exclusion viability method. The number of stained cells was subtracted from the total, indicating the death ratio in each interaction condition and time.

### Cell proliferation assay

Z172 were cultured in the low glucose DMEM and DMEM-YI-S medium (2:1, vol/vol) with supplied with 2% haemoglobin. Z172 were seeded to 24-well polypropylene culture plate, respectively. The cultures were incubated at 37°C, 5% CO2. After overnight, the medium was replaced and 50 ul of MTT (1mg/ml) was added to each well at 37°C and 5% CO_2_ for 4 h. After incubation, the cultures were removed the medium and addition DMSO. The absorbance was measured on ELISA Microwell Reader at 590/630 nm. Mann-Whitney U test was used for the significant test in the study.

### Viability test assay

Z172 cells were grown on 24-well of polypropylene culture plate and cultures were incubated at 37°C, 5% CO_2_. After parasites interaction with Z172 the medium was replaced and 50 μl of MTT (1 mg/ml) added to each well at 37°C and 5% CO2 for 4 hours. Cultures were removed the medium and addition DMSO for each sample. The absorbance was measured on ELISA Microwell Reader at 590/630 nm.

### Visualze cytoskeletal rearrangements in infected cells

Using the Lipofectamine 2000 for the transfection of nucleic acids of pAcGFP1-actin vector (green fluorescent protein and cytoskeleton targeting sequence) was applied for the transfection DNA, and we for the label of actin. The DNA mixture was then incubated at room temperature for an additional 30 min to allow complex formation. Z172 cells were washed twice with phosphate-buffered saline and DMEM without serum.

### Light and fluorescent

The image analysis is uses for the software of CellR (Olympus America, Inc., Melville, NY). It is used for the observation the changes of cells after *T*. *vaginalis* attacking.

### Flow cytometry

The propidium iodide (PI) intercalates into the major groove of double-stranded DNA and produces a highly fluorescent adduct that can be excited at 488 nm with a broad emission centred around 600 nm. It can be used to quantitate apoptosis by flow cytometry. The system was performed according to the protocol established by Riccardi and Nicoletti [29].

### RNA extraction

The total RNA of *T*. *vaginalis* and Z172 cells were extracted with TRIzol Plus RNA purification system reagent (Invitrogen) for the following experiment such as SuperArray and reverse transcription polymerase chain reaction (RT-PCR). Briefly, Z172 cells were seeded onto 10cm2 culture dish and allowed to form a monolayer for 2 days. Cells were washed with a medium mixture of DMEM-YI-S (2:1, vol/vol) without serum. Protozoa, which in the mid-logarithmic phase of growth, were added to the Z172 monolayer at Z172- *T*. *vaginalis* ratio of 1:10 and incubated at 37°C to allow for parasite adherence. Parasites were incubated with the Z172 cells within 12 hours, and non-adherent parasites were removed by aspiration. The detail procedures were processed according to the manufacturer’s protocol. The entire concentration and A260/A280 ratio of mRNA was measured with ND-1000 (NanoDrop).

### Reverse transcription PCR

One step reverse transcription PCR was performed with SuperScript One-Step RT-PCR with Platinum Taq kit (Invitrogen) to investigate the gene expression of *T*. *vaginalis* of ap65 [30]、β-tubulin and ap65-1 [31]. All the cDNA was synthesized from 1 μg of total RNA of *T*. *vaginalis* and Z172. RT-PCR product separated on EtBr-stained gel after electrophoresis in 1.0–1.2% agarose. The rest procedures were processed according to the manufacturer’s protocol. Primers were listed in S1 Table.

### Oligo GEArray

The Oligo GEArray Human Common Cytokines Microarray profiles (Cat. No. OHS-021) can be detected the expression of 114 important cytokine genes. Also represented are colony-stimulating factors and various growth factors (fibroblast, insulin-like, platelet-derived, transforming, and vascular endothelial). Tumor necrosis factors are included as well as other cytokine-related genes. Through a simple side-by-side hybridization experiment determine differential gene expression. The we add 5 μg aliquots of mRNA samples from Z172 cells by TrueLabeling primer and cDNA synthesis buffer and cDNA synthesis Enzyme mix (SABiosciences, USA) for cDNA synthesis. Next step for cDNA amplification were using Biotinylated-UTP and RNA polymerase buffer and RNA polymerase Enzyme. cRNA synthesis reaction and purification were using cRNA Cleanup Kit. The purified and concentrated cRNA for the experimental samples were mixed, and a hybridization mixture was prepared in GEAhyb Hybridization solution and hybridization tubes add cRNA target hybridization mix at 60°C, 5–10 rpm, in hybridization oven incubators, overnight. Chemiluminescent detection in 37°C pre-warm GEAblocking solution Q and 5X buffer at room temperature. Finally, discard buffer, repeat two times and rinse with 3 ml buffer G, invert three times and 1 ml CDP-star chemiluminescent substrate and detection by UV Photometer.

## Results

### Effects of adaptation of *T*. *vaginalis* and Z172 cells in co-culture medium

This study established a co-culture system to characterize the interaction between *T*. *vaginalis* and host cells. After adapting *T*. *vaginalis* from pH 5.8 to 7.2 in YIS medium, we added DMEM medium serially until reaching a volume ratio of DMEM-YIS (co-culture) medium of 2:1. No significant difference was observed in the morphology of *T*. *vaginalis* following twenty passages of adaptation (S1A and S1C Fig). To verify the effects of adaptation on the growth curve of parasites, we monitored the *T*. *vaginalis* by counting the number of cells stained with trypan blue solution in a neubauer hemocytometer chamber. The time required for the doubling of *T*. *vaginalis* were 4, 5.8, and 6.7 hours in pH5.8 YIS, pH7.2 YIS, and DMEM-YIS medium, respectively. The growth rate of *T*. *vaginalis* was slower in co-culture medium; however, the parasite density still reached 2x10^6^ cells per ml.

Z172 cells were also adapted from pH 7.2 DMEM to co-culture medium. Again, no difference was observed between the morphology (S1B and S1D Fig) or growth curves of Z172 cells in DMEM and the co-culture medium. Images were taken every 30 minutes using a differential interference contrast microscope to facilitate the long-term observation of Z172 cells in co-culture medium. Our results showed no effect on the growth of the cells (S2 Fig).

### Appropriate ratio of parasites and Z172 cells in co-culture system for the study of pathogenesis

**Fig 1:**
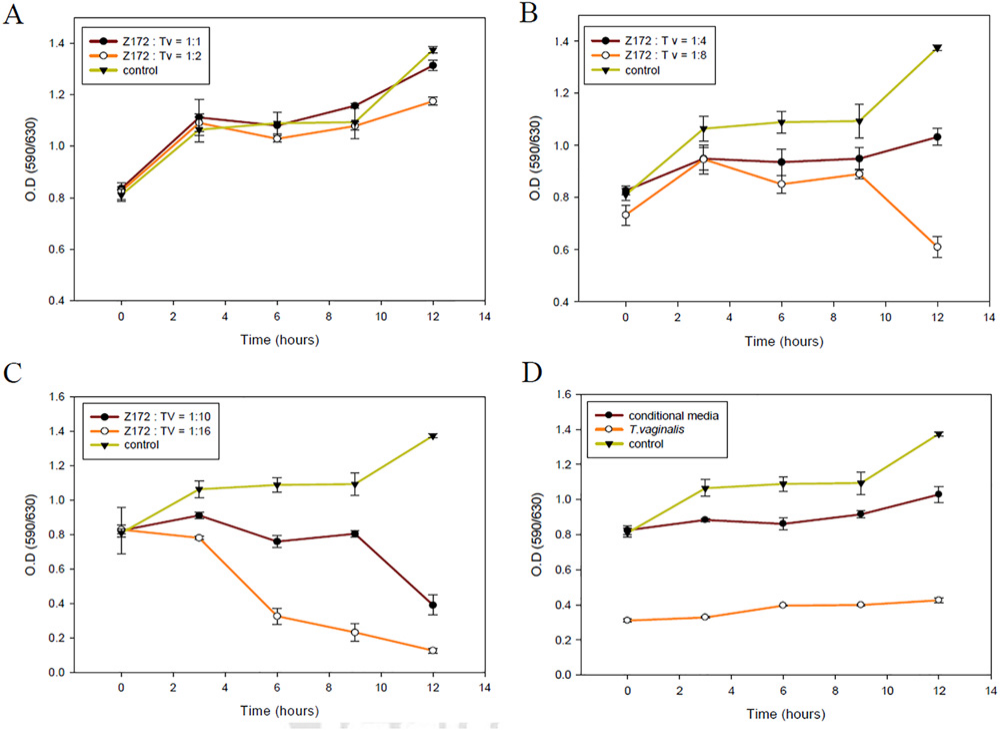
The cell viability of the co-cultured *T*. *vaginalis* and Z172 cells in different ratio. The cell viabilities of Z172 cells which were co-cultured with the ratio of parasite density, 1:1 and 1:2 (A), 1:4 and 1:8 (B), 1:10 and 1:16 (C), were tested by MTT assay during 12 hours. The conditional media means that Z172 cells were cultured with the medium ever cultured *T*. *vaginalis* (D).

**Fig 2:**
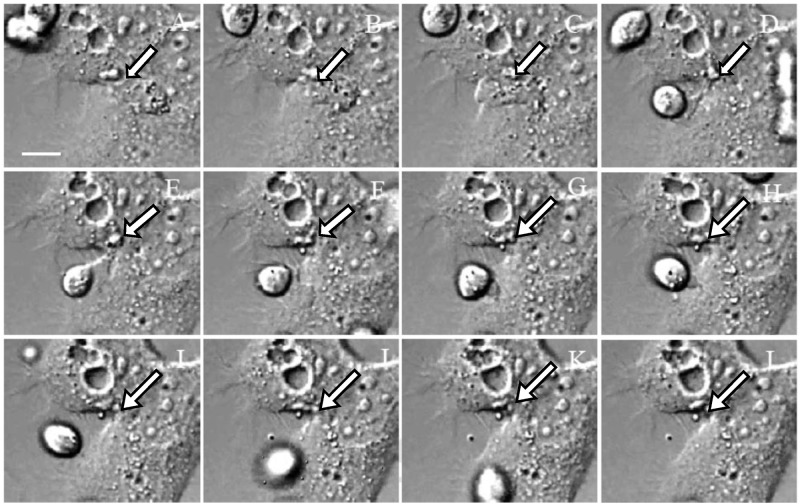
The light observation for the live Z172 cells imaging by time-lapse recording. The light observation (Differential interference contrast microscopy) of the progress of Z172 cells which was transfected pAcGFP1-Actin after co-cultured with *T*. *vaginalis* in 1:10. Panel A to L were the captured images once every 30 minutes. The arrows indicate the position of the cell gap.

To determine the interaction level of *T*. *vaginalis* with Z172 cells, we performed a preliminary experiment using various parasite densities (1:1, 2:1, 4:1, 8:1, 10:1 and 16:1). Parasites interacting with Z172 cultures were monitored once every 2 h for the first 12 h. Our results showed that at a parasite:Z172 ratio of 1:1 or 2:1, the growth of Z172 cells remained unaffected (Fig 1A). When the parasite:Z172 ratio was increased to 4:1, 8:1, or 10:1, the growth of Z172 cells slowed slightly (Figs 1B and 2C). When the ration was increased to 16:1, cellular damage was too extensive to allow for further analysis (Fig 1C). Thus, we selected a parasite:Z172 ratio of 10:1 for subsequent experiments.

### Morphological changes in Z172 cells caused by adherence of *T*. *vaginalis*

**Fig 3:**
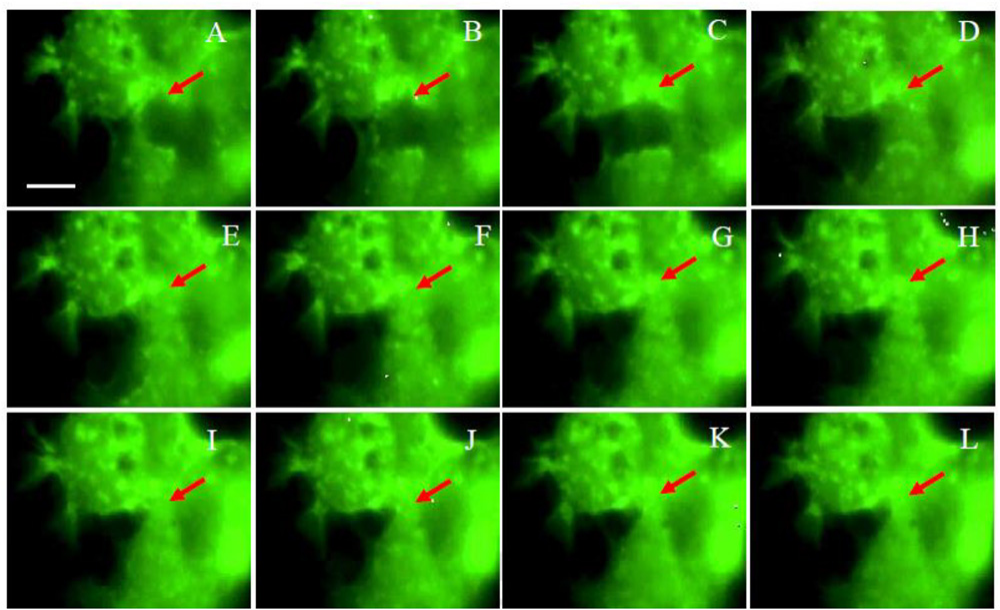
The fluorescence observation for the live Z172 cells imaging by time-lapse recording. The fluorescent observation of the progress of Z172 cells which was transfected pAcGFP1-Actin after co-cultured with *T*. *vaginalis* in 1:10. Panel A to L were the captured images once every 30 minutes. The arrows indicate the position of the cell gap.

To identify the morphological changes in Z172 cells attacked by T. vaginalis, this study transfected pAcGFP-actin into Z172 cells co-cultured with parasites. Images were taken every 30 minutes using a differential interference contrast microscope to facilitate the long-term observation of Z172 cells in co-culture medium (Fig 2) and fluorescence microscope (Fig 3). These results clearly indicate that the attachment of *T*. *vaginalis* initiated damage to the Z172 cells. As incubation time was extended, the adhesion of *T*. *vaginalis* induced changes in the shape of Z172 cells and an increase in the distance between cells. These results prove once again that the cytotoxicity of *T*. *vaginalis* begins at the time of initial attachment. The adhesion of *T*. *vaginalis* induced death in the host cell as well as the destruction of the cell-cell junction, which triggered a disruption to the epithelial cells. We also prolonged the monitoring period in order to observe Z172 cells cultured with inactive *T*. *vaginalis* (S3 Fig) as well as those in parasite cultured medium (S4 Fig). The results of these experiments showed that only living *T*. *vaginalis* induced damage through contact.

**Fig 4:**
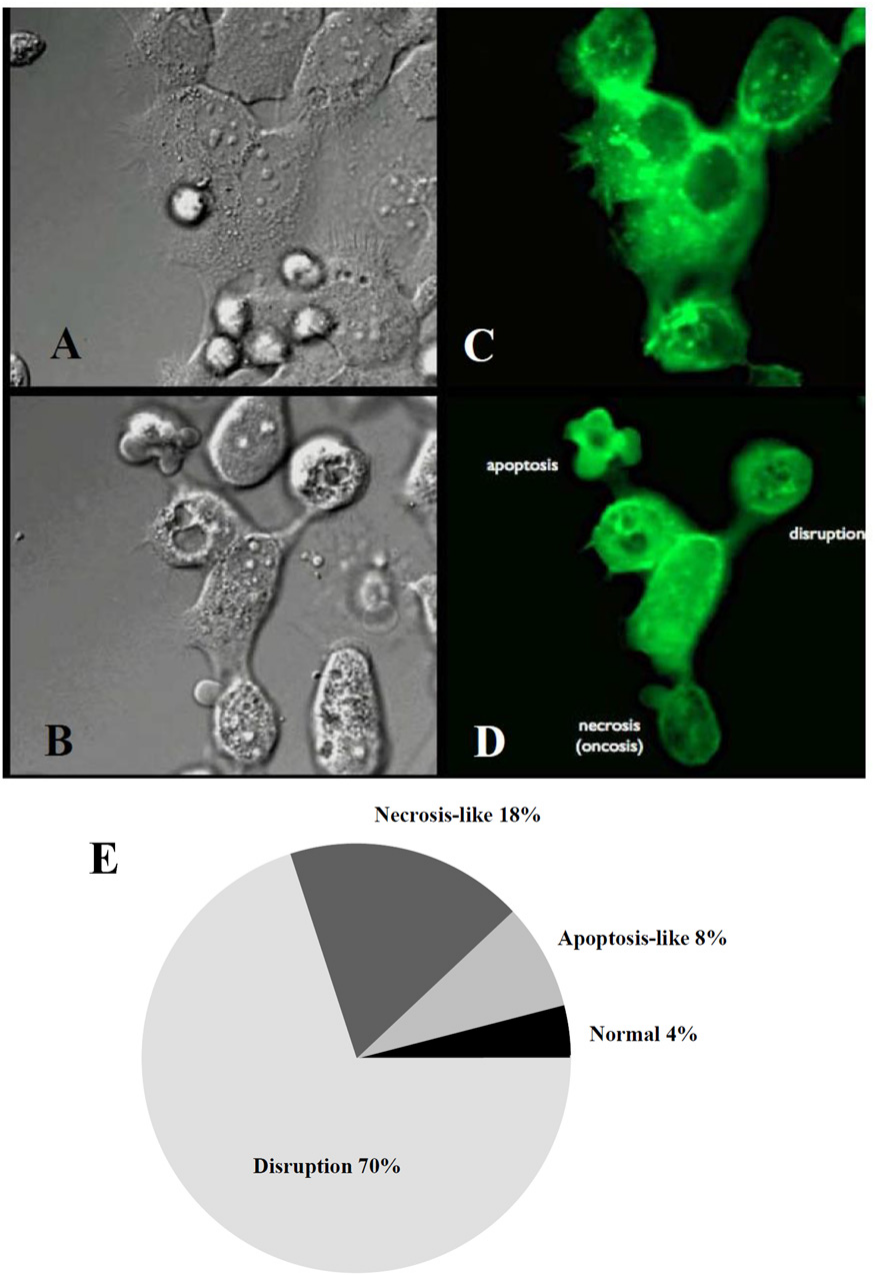
The morphological change of Z172 cells after co-cultured with *T*. *vaginalis*. Panel A and B were light observed before and after co-cultured 10 hours, Panel C and D were captured in fluorescent observation. Bar = 10 μm. The statistical results of the cell morphological changes were shown in panel E.

Following co-culturing with *T*. *vaginalis* for 10 hours, three types of cell morphological changes were observed in the Z172 cells: disruption, necrosis-like cell death, and apoptosis-like cell death (Fig 4B and 4D; S1–S6 Video). Time-lapse photography was used to record changes in the shape of Z172 cells in order to facilitate further analysis and statistical observation, the results of which showed that 70% of cells had undergone disruption, 18% of cells were necrosis-like, 8% of cells were apoptosis-like, and 4% of cells did not present significant morphological changes (Fig 4E). These results lead to the conclusion that after being attacked by T. vaginalis, Z172 cells shrink, become more spherical, and the gap between the cells becomes larger, but the main mode of disruption is by way of cell death.

### Cell cycle phase of Z172 cells co-cultured with *T*. *vaginalis*

This study used the PI staining of DNA to study the effects of *T*. *vaginalis* on the cell cycle phase of Z172 cells. The results were then confirmed by flow cytometry showing that most of the Z172 cells were arrested in the G^0/1^ phase and a small fraction in the sub-G^0/1^ phase. No significant differences in Z172 cell cycle phase profiles were observed after further interaction between parasites and Z172 cells (S5 Fig). This suggests that the cell damage caused by *T*. *vaginalis* is unrelated to cell cycle progression.

### Increased RNA expression levels in *T*. *vaginalis AP65* and *AP65-1* with co-culture time

**Fig 5:**
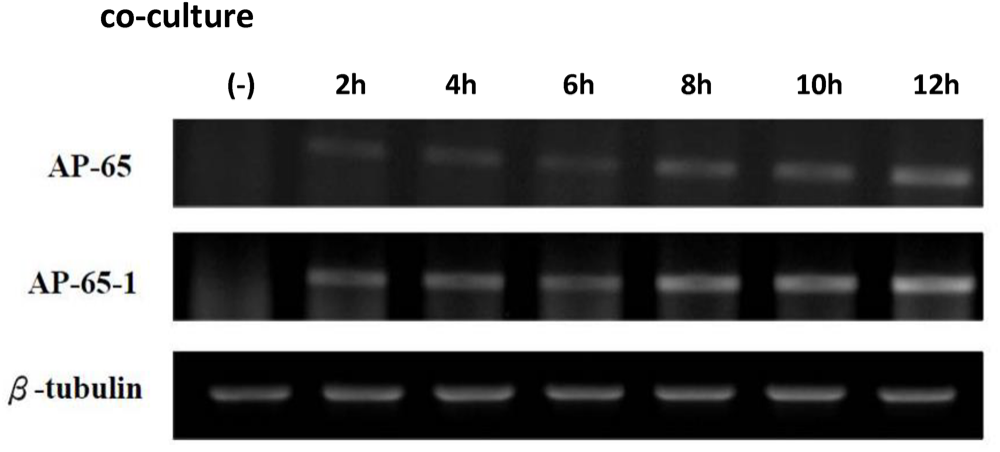
The gene expression of *T*. *vaginalis* which co-cultured with Z172 cells. The changes of gene expression levels of *T vaginalis* after interacted with Z172 cells were investigated by RT-PCR. The RT-PCR products separated on EtBr-stained gel after electrophoresis in 1.0–1.2% agarose. lane 1:AP-65, lane 2:AP-65-1 and lane 3:β-Tubulin。

We next surveyed differences in the gene expression of parasites and host cells throughout the co-culture process. We used primers specific to *ap65* and *ap65-1*of *T*. *vaginalis* in order to detect the RNA expression levels of the adhesion proteins by RT-PCR. Our results showed that the expression levels of *ap65* and *ap65-1* in *T*. *vaginalis* increased with an extension of co-culture time (Fig 5).

### Changes in gene expression profile of co-cultured Z172 cells

**Fig 6:**
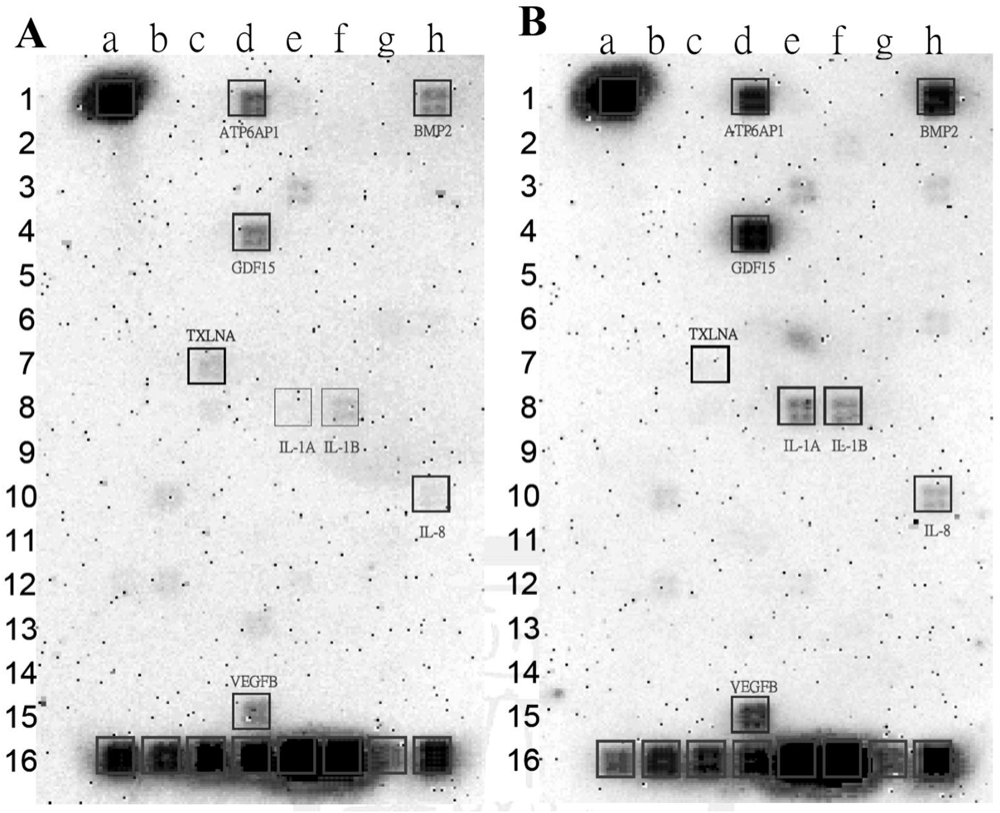
The cytokine genes expression profile of Z172 cells which co-cultured with *T*. *vaginalis*. The changes of gene expression levels of Z172 cells before (A) and after interacted with *T vaginalis* (B) were investigated by Oligo GEArray Human Common Cytokines Microarray.

In order to clarify the effects on gene expression in human cells attacked by *T*. *vaginalis*, we analyzed the gene expression profiles of Z172 cells using the Oligo GEArray Human Common Cytokines Microarray. A total of 114 important cytokine genes, colony-stimulating factors, tumor necrosis factors, various growth factors, and the other cytokine-related genes are covered by this chip. Our results revealed seven cytokine associated genes concomitant with an increase in expression levels and one associated with a decrease (Fig 6). The down-regulated gene was Taxilin-α, TXLNA (c-7) and the up-regulated genes were bone morphogenetic protein 2, BMP2 (h-1); growth differentiation factor 15, GDF 15 (d-4); IL-8, (h-10); IL-1αand IL-1β, (e-8 and f-8); ATP6AP1 (d-1); and vascular endothelial growth factor B, VEGF-β (d-15). These genes play important roles in the immune response, inflammatory response, and cell-cell signaling.

## Discussion

*Trichomonas vaginalis*, an ancient protozoon, is the causative agent in one of the world's most common sexually infected disease, with a worldwide infection rate of 170 million annually [32]. Infection is acquired primarily through direct sexual contact although neonatal infection has also been reported [33–35]. *T*. *vaginalis* colonizes and parasites in the vagina or prostate of the infected host cause trichomonosis that can lead to severe health complications. Previous reports have shown that *T*. *vaginalis* infection results in cervical epithelial cell death via a strictly contact-dependent mechanism [11].

The importance of maintaining an intact mucosal layer is highlighted by the occurrence of pathological disorders associated with inflammatory disease, in which disruption of the epithelial barrier leads to severe inflammation of the mucosal tissue compartments [17, 36, 37]. The epithelial barrier is maintained by connections between adjoining epithelial cells, which are the first sites of contact with the host. These cells are responsible for separating potentially harmful luminal content from the underlying tissue. This function of acting as a physical barrier is accomplished by junctional complex, which comprises a plethora of membrane-associated and transmembraneous proteins organized in discreet, spatially restricted complexes [37, 38]. Our findings provide solid evidence to support claims that the damages of *T*. *vaginalis* infection to host cells and cell junctions is initiated by the attachment of live parasites and cannot be induced by the inactivated *T*. *vaginalis* or parasite cultured medium. Previous reports have shown that host cells infected by *T*. *vaginalis* presented disruption before being lysed [10, 39]. In addition to damage and disruption in the cells, we also observed in time-lapse recording apoptosis-like and necrosis-like cell death of the Z172 cells co-cultured with *T*. *vaginalis* for ten hours.

Epithelial cells act in a manner similar to that of a connector in a host-microbe communications network, building signal transduction connections between luminal microbes and the host. The epithelial cells act as sensors for the microbes and as providers of signal to immune cells to activate inflammatory and immune responses. The infection accompanied by the release of cytokines and chemokines is caused by inflammation of one of the causes. The array results in this study showed an increase in the expression of seven cytokine-associated genes following infection with *T*. *vaginalis* in Z172. In these cytokines, IL-8 is known to govern polymorphonuclear leukocytes localization and function. Shaio reported the interleukin-8 response to infection with *T*. *vaginalis* in human monocyte and neutrophils [40, 41]. In T. vaginalis-infected human ectocervical cells, parasites as well as exosomes were shown to specifically modulate IL-8 [42]. Interestingly, Twu et al. reported that *T*. *vaginalis* produces and secretes exosomes capable of promoting parasite:parasite communication and host cell colonization, as well as mediating the immune response of host cells [42]. In this study, we did not observe any effects of Z172 with the medium with which parasites were cultured. We propose the following explanations: Either the release of exosomes must be induced or the concentration of exosomes is too low to induce any visible effects.

Previous reports have shown that Entamoeba histolytica co-cultured with human intestinal epithelial cells increase the expression and secretion of chemoattractant and proinflammatory cytokines, including IL-8, GROα, GM-CSF, IL-lα, and IL-6 [43]. We also found that proinflammatory cytokines, IL-1α and IL-1β, are induced after the attachment of T. vaginalis. The secretion of IL-1β into surrounding interstitial fluid and blood during inflammation mediates a wide range of proinflammatory activities. IL-1β has been shown to cause a significant increase in intestinal tight junction permeability [44]. Wang et al. reported that VEGF could induce tight junction disassembly and alter the distribution of actin filaments in microvessel endothelial cells in the brain by reducing ZO-1 and occludin located at tight junctions [23].

We also found that VEGF-β is induced in human ectocervical cells infected with T. vaginalis. According to these results, it is reasonable to assume that the disruption of host cell pathogenesis may be caused by these cytokines. Another cytokine which increased expression level, BMP-2 is a member of the TGF-β superfamily, playing an important role in the embryonic development, suppression of the immune response, and differentiation and proliferation of tissues and cells. In mature colonic epithelial cells, it acts as a tumor suppressor capable of promoting apoptosis [45]. We propose that the death of Z172 not caused by infection, but hyperimmunization of cytokines.

In summary, we hypothesize that the morphological and gene expression of Z172 is altered to form cytokines and a complex signaling cascade in response to trichomonal adherence. During the parasite and host cell interaction, *T*. *vaginalis* as well as Z172 cells stimulate each other to induce the differential expression of genes. Contact-dependent changes in cytotoxicity of human cervical cells are important in the pathogenesis of human cervical disease and may also be an effective target for the development of new drugs.

## Supporting Information

S1 Fig The morphology of the *T*. *vaginalis* and Z172 cells before and after adaptation. The *T*. *vaginalis* and Z172 cells were adapted respectively from the original medium, the YI-S medium (panel A) and the DMEM medium (panel B), into the co-culture medium, DMEM:YI-S (2:1, vol/vol) medium (panel C and D). The adapted *T*. *vaginalis* and Z172 cells were co-cultured in DMEM:YI-S medium (panel E). Bar = 20μm.
(TIFF)

S2 Fig The morphology of the Z172 cells were cultured in DMEM:YI-S (2:1, vol/vol) medium for the long-term observation. Panel A to T were the captured images once every 30 minutes. Bar = 20μm.
(TIFF)

S3 Fig The morphology of the Z172 cells were cultured in the DMEM:YI-S medium which have been cultured *T*. *vaginalis*. Panel A to T were the captured images once every 30 minutes. Bar = 20μm.
(TIFF)

S4 Fig The morphology of the Z172 cells were cultured in the DMEM:YI-S medium with the inactived *T*. *vaginalis*. Panel A to T were the captured images once every 30 minutes. Bar = 20μm.
(TIFF)

S5 Fig Percentages of the Z172 cells which were co-cultured with *T*. *vaginalis* were in different phases of the cell cycle. The propidium iodide (PI) was used to stain the DNA and look for the sub-diploid to quantitate apoptosis by flow cytometry.
(TIFF)

S1 Table Primer list of *T*. *vaginalis* used for PCR and RT-PCR reactions. (DOCX)

S1 Video Disruption (Nomaski). The observation of the progress of Z172 cells which co-cultured with *T*. *vaginalis*.
(MOV)

S2 Video Disruption anti-actin (GFP). The observation of the progress of Z172 cells which co-cultured with *T*. *vaginalis*.
(MOV)

S3 Video Apoptosis (Nomaski). The observation of the progress of Z172 cells which co-cultured with *T*. *vaginalis*.
(MOV)

S4 Video Apoptosis anti-actin (GFP). The observation of the progress of Z172 cells which co-cultured with *T*. *vaginalis*.
(MOV)

S5 Video Cytolysis (Nomaski). The observation of the progress of Z172 cells which co-cultured with *T*. *vaginalis*.
(MOV)

S6 Video Cytolysis anti-actin (GFP). The observation of the progress of Z172 cells which co-cultured with *T*. *vaginalis*.
(MOV)
