# Supplementary figures and images for: The Pathogenesis of Human Cervical Epithelium Cells Induced by Interacting with Trichomonas vaginalis
Source: PLoS One. 2015 Apr 22;10(4):e0124087. doi: 10.1371/journal.pone.0124087 (PMC4406492; doi:10.1371/journal.pone.0124087)

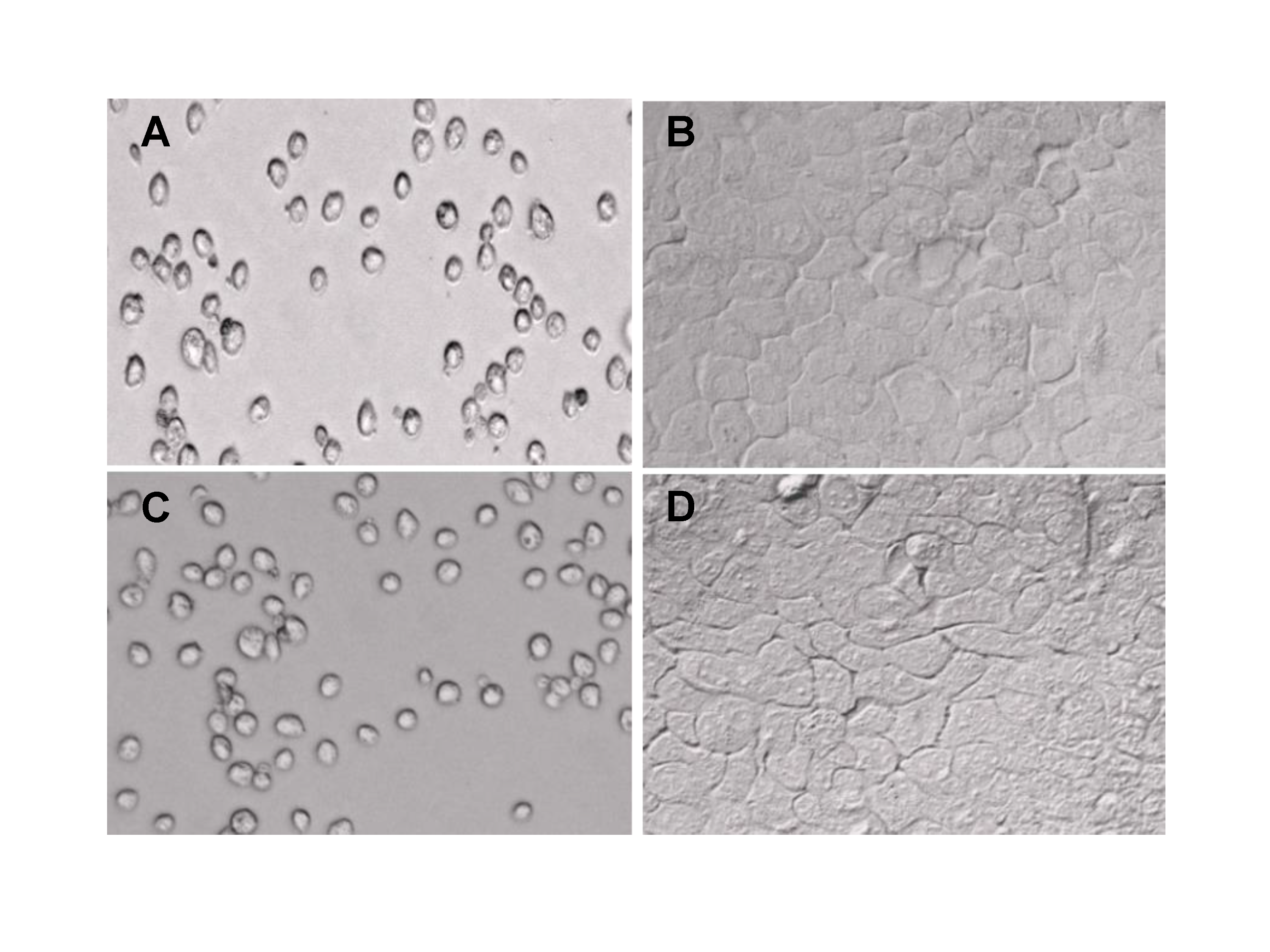

Supplement: S1 Fig — The T. vaginalis and Z172 cells were adapted respectively from the original medium, the YI-S medium (panel A) and the DMEM medium (panel B), into the co-culture medium, DMEM:YI-S (2:1, vol/vol) medium (panel C and D). The adapted T. vaginalis and Z172 cells were co-cultured in DMEM:YI-S medium (panel E). Bar = 20μm. (TIFF) [file pone.0124087.s001.tiff]

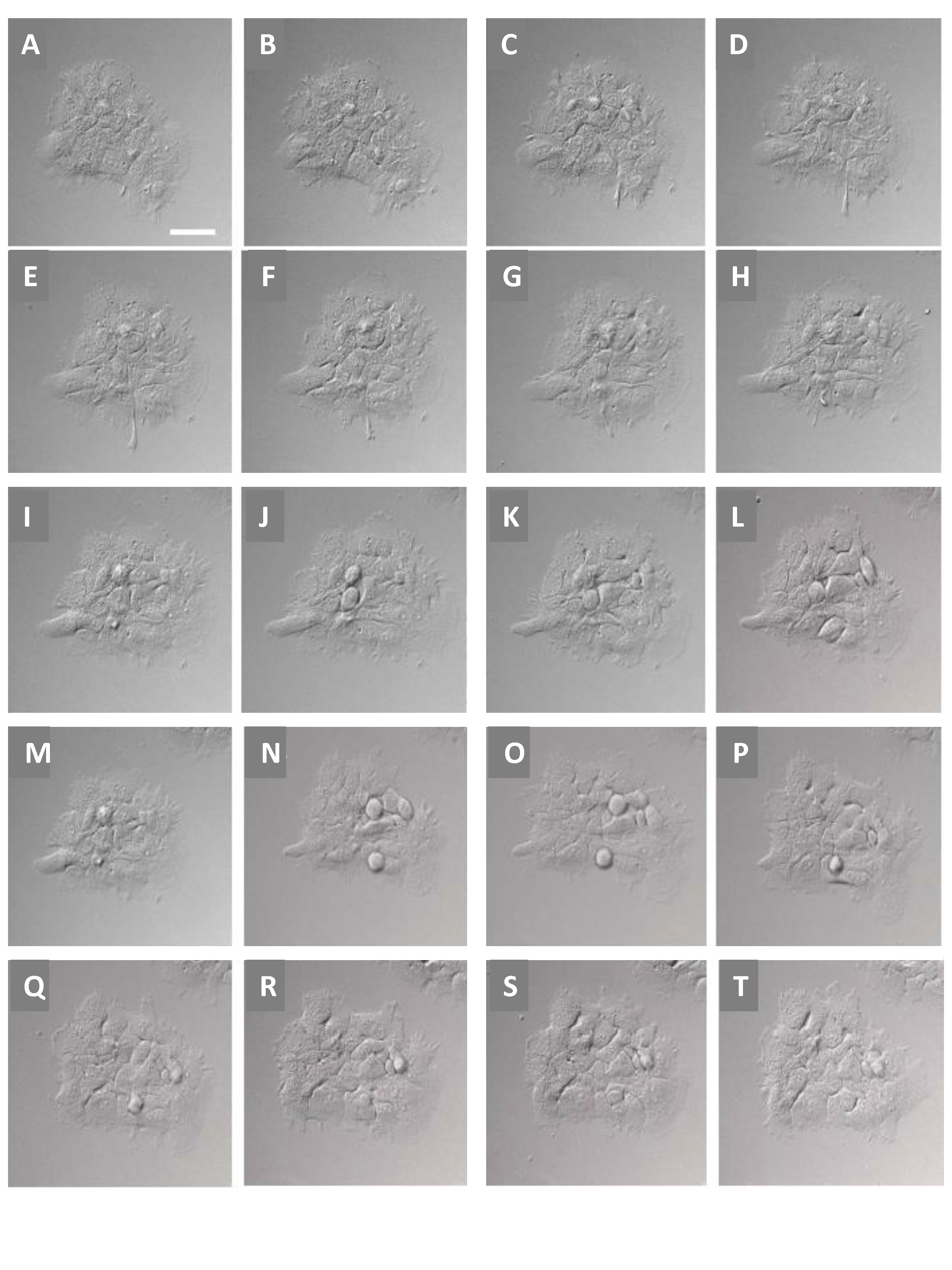

Supplement: S2 Fig — Panel A to T were the captured images once every 30 minutes. Bar = 20μm. (TIFF) [file pone.0124087.s002.tiff]

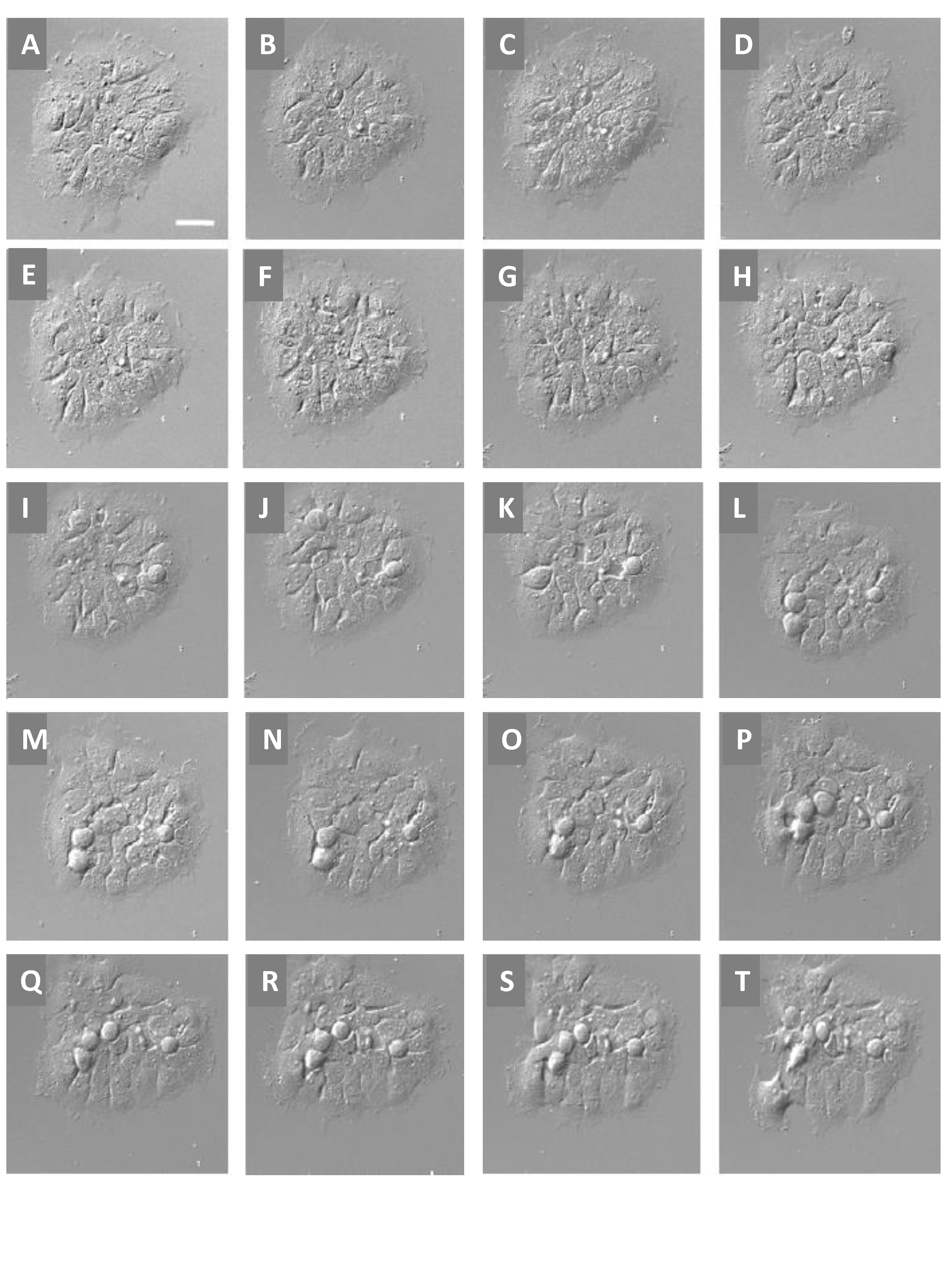

Supplement: S3 Fig — Panel A to T were the captured images once every 30 minutes. Bar = 20μm. (TIFF) [file pone.0124087.s003.tiff]

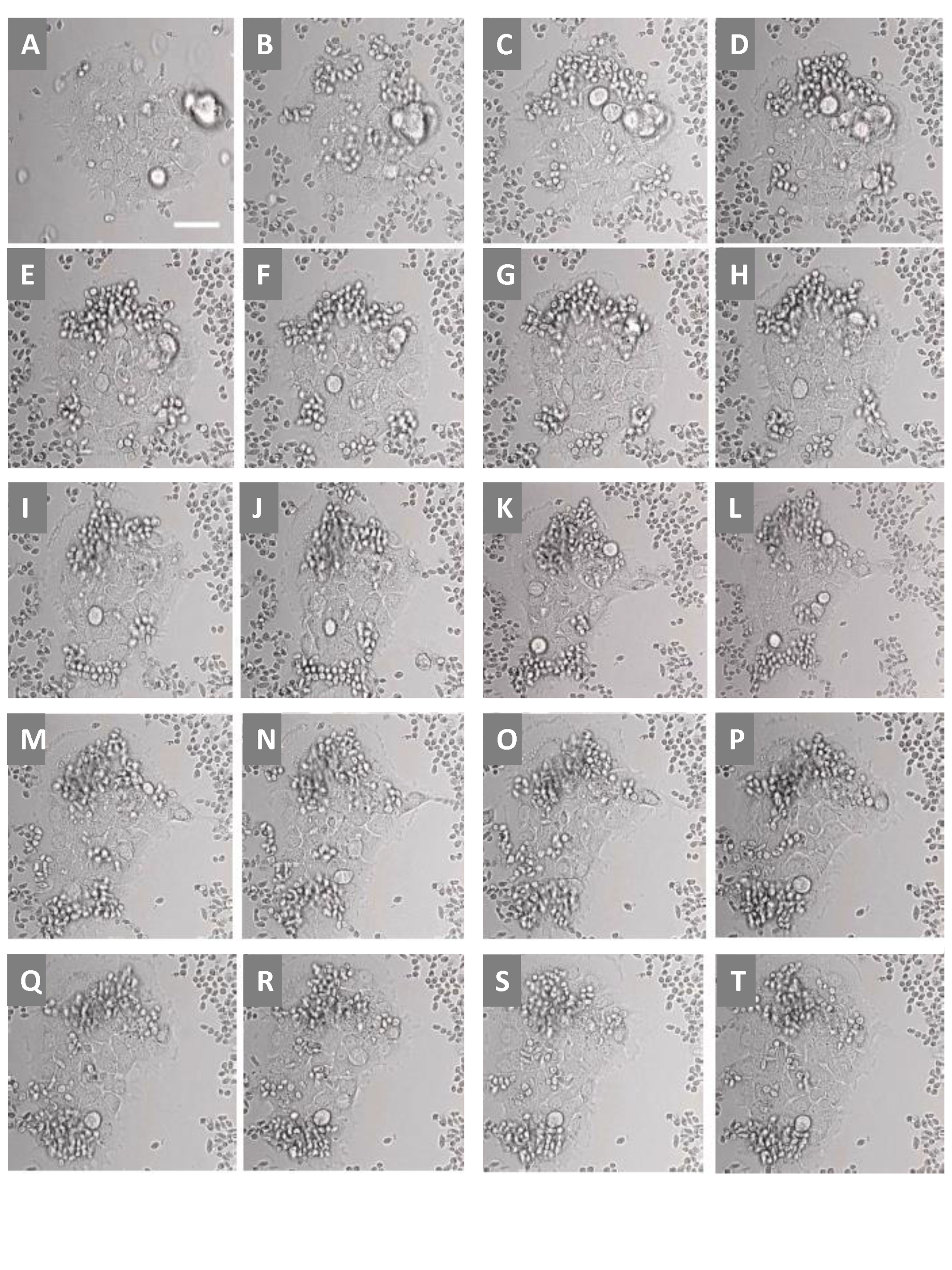

Supplement: S4 Fig — Panel A to T were the captured images once every 30 minutes. Bar = 20μm. (TIFF) [file pone.0124087.s004.tiff]

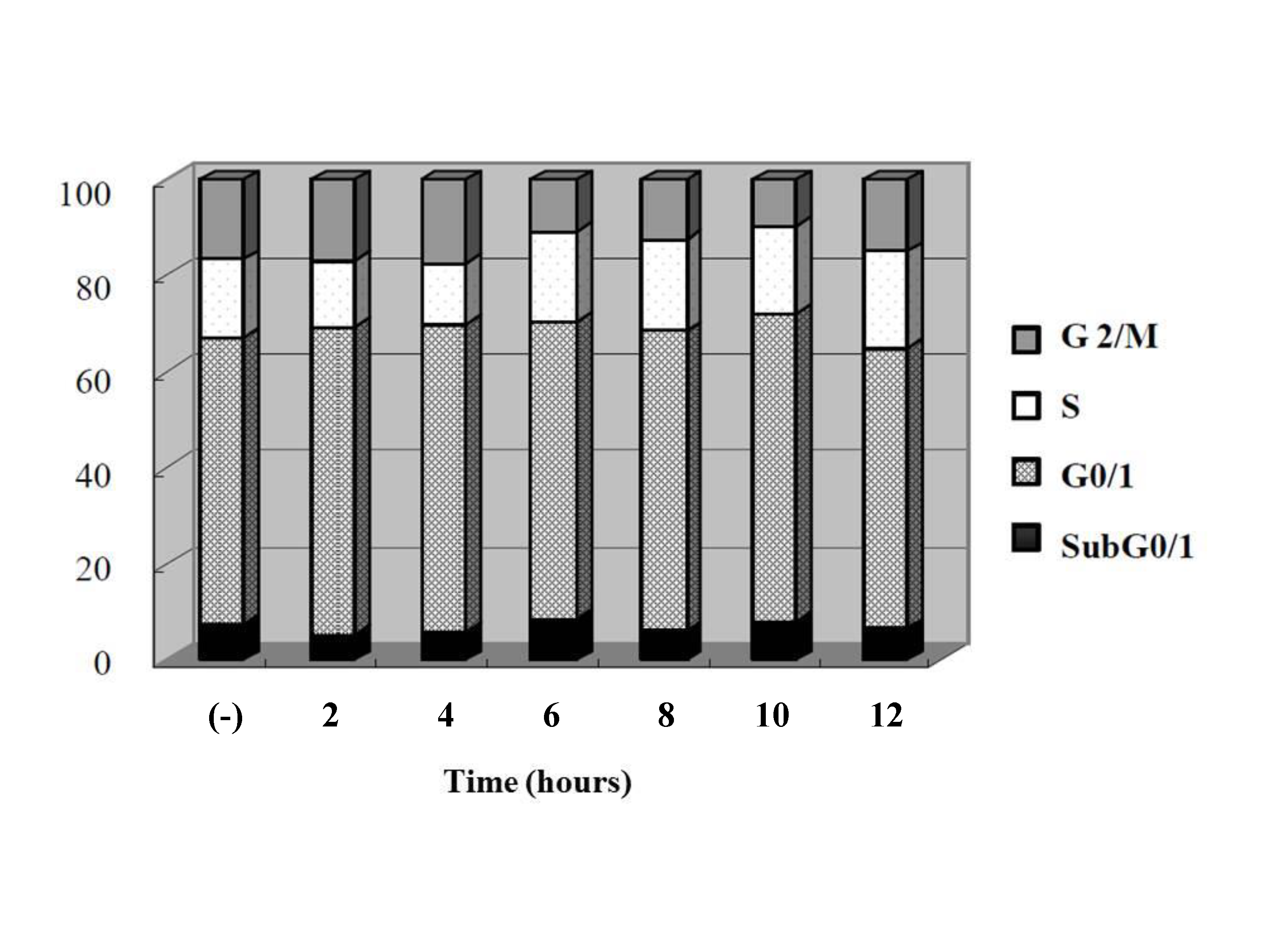

Supplement: S5 Fig — The propidium iodide (PI) was used to stain the DNA and look for the sub-diploid to quantitate apoptosis by flow cytometry. (TIFF) [file pone.0124087.s005.tiff]
