# Supplementary material for: The Pathogenesis of Human Cervical Epithelium Cells Induced by Interacting with Trichomonas vaginalis
Source: PLoS One. 2015 Apr 22;10(4):e0124087. doi: 10.1371/journal.pone.0124087 (PMC4406492; doi:10.1371/journal.pone.0124087)
Supplement: S1 Table — (DOCX) [file pone.0124087.s006.docx]

| Accession | Annotation | Primer sequence | Product size (bp) | Reference |
| --- | --- | --- | --- | --- |
| U 18346.1 | AP65-1 adhesion | 5’- AATggCAAggCCCTCTgCgCTAC-3’  5’- TAAATTAAgAAAgCTAAgTgTTTAAAAATCgCgC-3’ | 430 | (30) |
| U 18346.1 | AP65 adhesion | 5’- gATTCCTCTTCACA ACCCACCAg -3’  5’- AATACggCCAgCATCTgTAACgAC -3’ | 209 | (29) |
|  | β -tubulin | 5’- AAATCgTTCACATCCAAgCTgg -3’  5’- TTgTATggCTCgACgACTgTATCAG -3’ | 545 | (30) |
